# Ecology of Freedom: Competitive Tests of the Role of Pathogens, Climate, and Natural Disasters in the Development of Socio-Political Freedom

**DOI:** 10.3389/fpsyg.2018.00954

**Published:** 2018-06-12

**Authors:** Kodai Kusano, Markus Kemmelmeier

**Affiliations:** Interdisciplinary Social Psychology Ph.D. Program, University of Nevada, Reno, NV, United States

**Keywords:** ecology, culture, pathogens, climate, natural disaster, freedom, individualism, democracy

## Abstract

Many countries around the world embrace freedom and democracy as part of their political culture. However, culture is at least in part a human response to the ecological challenges that a society faces; hence, it should not be surprising that the degree to which societies regulate the level of individual freedom is related to environmental circumstances. Previous research suggests that levels of societal freedom across countries are systematically related to three types of ecological threats: prevalence of pathogens, climate challenges, and natural disaster threat. Though their incidence overlaps, the literature has not yet provided a competitive test. Drawing upon the ecocultural framework, we tested five rival hypotheses, alternately focused on the above ecological factors and their interactions with economic wealth in explaining country variations in socio-political freedom. Focusing on data from 150 countries, we performed a series of linear mixed-effects regressions predicting freedom in the domains of politics, media, and economy. We found that countries with higher pathogen prevalence were more likely to suppress democracy and media freedom. Economic wealth, however, moderated the effect of pathogen prevalence on economic freedom, with the main effect being only found among wealthy countries, but not among poor countries. In contrast, natural disaster threat predicted political freedom and press freedom only among poor countries, consistent with the idea that disaster threat accompanied by poor resources promote socio-political freedom as a means of increasing collective survival. Throughout our analyses, we found no support for hypotheses based on climatic challenges. In addition, our multilevel approach revealed that country scores for socio-political freedom were highly clustered within world regions, accounting for substantial portions of variance. Overall, the present research offers a nuanced view of the interplay between ecology and wealth in the emergence of socio-political freedom. We discuss new directions in future research considering methodological and theoretical contributions of the present findings.

## Introduction

Freedom is a central tenet of democracy. It is central to many founding documents of democratic states, whether it is the Magna Carta, U.S. Declaration of Independence, or the constitutions of most democratic countries. While all countries regulate the amount of freedom given to their citizens through various socio-political institutions such as politics, media, and economic infrastructures, democracies vary in the kind and extent of the freedom they grant their citizens. Moreover, democracy itself is not universal, with many societies struggling or regimes abandoning the advancement of democratic freedoms. But why do some countries bestow great freedom, while other countries seem to repress it? In this study, we explore this question and the broader conditions of when and where freedom takes a hold within socio-political structures from the ecocultural framework (Berry, 2002).

Research from various disciplines of the social sciences have proposed theories to explain systematic country-variations in cultural systems and social structures. Of them, an emerging body of research now suggests that ecological conditions might be antecedents to the level of societal freedom. Previous research in the ecocultural framework has shown that three ecological conditions—pathogens, climate, and natural disasters—appear to affect the levels of socio-political freedom. Despite the findings consistently supported by each theory, there has been little effort to test these theories against each other. Therefore, a major goal of this study was to provide a competitive test of existing hypotheses concerning contemporary cross-country variations in the levels of socio-political freedom.

### Ecocultural hypothesis of country-variations in socio-political freedom

Characteristics of cultures and socio-political structures are inextricable from the nature of environments upon which they are built. Theories examining the close relationship between ecology and cross-cultural differences dovetail with Berry's (2002) ecocultural framework, which conceptualizes culture as a group-level adaptation to given ecological conditions. According to various theorists (Fehr et al., 2002; Boyd and Richerson, 2009), the prime strategy to deal with adversity among humans is cooperation—a mechanism that allows the flexible combination of the effort of multiple individuals for mutual benefit. Case studies, experiments, and simulations have typically demonstrated that individuals embedded in cooperative groups are much more likely to survive invasion or resource challenges than individuals acting by themselves, even though cooperation does impose a cost on the individual (e.g., see Axelrod and Hamilton, 1981).

The creation of culture reinforces this cooperative cycle. Culture facilitates social coordination by providing a set of principles that allow a group to solve basic needs of survival (Matsumoto and Juang, 2013). Thus, the development of culture results in greater efficiency and productivity of a group. From the ecocultural perspective, institutions also play an essential role of permeating unique cultural values to the majority of members within each cultural unit by legalizing cultural practices^1^. However, how a culture organizes a group depends on the types of ecological conditions that pose specific needs and problems; different types of cultures (institutions) emerge through the process by which people respond to different types and degrees of ecological threats across a wide range of regions (Tooby and Cosmides, 1992). Therefore, cross-country variations in the degrees of socio-political freedom reflect the types of ecological conditions that evoke different adaptive responses. Consistent with this general framework, researchers have submitted several hypotheses to explain what and how ecological conditions affect societal freedom. In the following section, we present reviews of major theories concerning pathogens, climate, and natural disasters.

#### Pathogens

Pathogens have been significant threats to humans throughout evolutionary history (Wolfe et al., 2007). Consequently, research considers pathogens as a major ecological threat that has significantly shaped human cultures throughout the history. A prevailing theoretical approach proposes that social interactions are characterized by the process by which people have adapted to pathogen stress (Fincher et al., 2008; see Thornhill and Fincher, 2014 for a related argument concerning parasite stress). The central assumption of pathogen-stress theory is that humans have developed psychological tendencies to detect and avoid infections in physical environments including human interactions (Schaller and Park, 2011). As human groups are likely to develop immune systems specific to local pathogens, contacts with out-groups are a major source of new pathogens (Thornhill and Fincher, 2014). Thus, people in regions with greater pathogen risks have greater incentives to avoid out-group contacts and favor in-group members. As a result, such regions are more likely to be characterized by collectivism, a tightly-knit community with sharp distinctions between in-groups and out-groups. However, communities with lower pathogen prevalence embrace individualism, maximizing individual freedom over in-group common goods for exploration.

Studies built upon pathogen-stress theory have empirically shown that collectivistic values are more prevalent in regions with greater pathogen prevalence; in contrast, in regions with lower pathogen prevalence, the benefits of out-group contacts—exchanges of new technologies, expansion of trading, and increased social alliances—outweigh its cost (Fincher et al., 2008; Murray et al., 2011). Therefore, pathogen-stress theory has much to offer for the explanation of cross-country variation in socio-political freedom. Several studies exploring this association found that countries with higher pathogen prevalence had lower levels of democracy, higher authoritarianism, as well as a higher degree to which governments restrict economic activities (Thornhill et al., 2009; Murray et al., 2013). Taken together, pathogen-stress theory predicts that higher pathogen prevalence is related to lower levels of socio-political freedom (Hypothesis 1).

#### Climate

Climate is another ecological threat that affects human societies. A considerable body of research on the effects of climate on human societies has been inspired by Van de Vliert's climato-economic theory (Van de Vliert, 2009). Climato-economic theory asserts that beyond pathogen prevalence, climates have critical implications for the function of human cultures and societies. The theory assumes that climate deviating from the comfortable temperature zone—above and below 22*C* (72°F)—requires physiological and psychological adaptations at the group level (Van de Vliert, 2006, 2011b). In general, societies in harsher climates (i.e., very hot or very cold) tend to repress societal freedom as a means of regulating individual freedom for collective survival. However, the availability of economic resources often mitigates the impact of climate: climate becomes a positive, stimulating challenge in societies with sufficient economic resources (Van de Vliert and Postmes, 2012). As a result, wealthy societies perceive climatic demands as an opportunity to pursue risk-taking, personal growth, self-expressions, and individual freedom (Van de Vliert, 2007).

A study guided by climato-economic theory found that wealthier countries accompanied by harsher climate strive for more liberal, democratic societies, whereas poorer countries with harsher climate exhibit relatively collectivistic values such as security for their own survival (Van de Vliert, 2013a). Germane to the present research, media repression was highest among poorer countries with harsher climate, whereas it was the lowest among wealthier countries with harsher climate (Van de Vliert, 2011a). Overall, the central prediction of climato-economic theory is that climatic demands are associated with higher levels of socio-political freedom among wealthy countries, whereas such an association is negative among poor countries (Hypothesis 2).

#### Natural disasters

Natural disasters like floods, storms, and earthquakes pose collective threats to people, and the consequences range from economic damage (Karim and Noy, 2016) to psychological distress (Nolen-Hoeksema and Morrow, 1991). Despite the enormous impacts on social and psychological functioning, theories and empirical studies on natural disasters generate mixed predictions regarding the role of natural disasters on the levels of socio-political freedom.

##### The contradictory role of natural disasters on socio-political freedom

The socio-ecological approach (Oishi and Graham, 2010), for example, broadly contextualizes ecology in the construction of human minds and socio-political structures. Previous research from this approach demonstrated that natural disaster risk was associated with higher levels of collectivism (Oishi and Komiya, 2017). Other work inspired by the socio-ecological approach found that prosocial values and behaviors significantly increased after major earthquake incidents in Japan (Oishi et al., 2017). This line of research assumes that natural disasters bring people together, or enhance collectivistic values, to mitigate the consequences of natural disasters. Therefore, these studies indicate that countries with a higher frequency or risk of natural disaster are associated with lower levels of socio-political freedom (Hypothesis 3).

Other studies, however, suggest that natural disasters drive individualism, a cultural phenomenon closely associated with individual freedom in various domains of social life (e.g., Triandis, 1995; Inglehart and Oyserman, 2004). For instance, a longitudinal study indicated that increases in disaster frequency preceded the rise of U.S. individualism as indexed by parents' preferences for unique baby names (Grossmann and Varnum, 2015). Similarly, another longitudinal analysis of 77 countries have implied that the temporal increase in disaster frequency preceded the rise of individualistic socioeconomic structures (Santos et al., 2017). These researchers interpreted the above findings as support for the social orientation hypothesis (Varnum et al., 2010).

According to this hypothesis, natural disasters lead to individualistic societies for two reasons: (1) Anxiety-provoking threats without an option to escape would narrow attentional and perceptual scope (Wachtel, 1968); and (2) Narrowed attention is an exemplary characteristic of the analytic thinking style, which is more frequently used by individualists than collectivists (Nisbett et al., 2001)^2^. Hence, natural disaster stress presumably elicits more use of analytic thinking styles among people, who might, in turn, use these cognitive styles to create individualistic, liberal, and democratic societies. According to the social orientation hypothesis, countries with a higher frequency of natural disasters tend to have higher levels of socio-political freedom (Hypothesis 4).

### Limitations in previous research

The theories introduced in the preceding section emphasize pathogens, climatic demands, and natural disasters as primary factors contributing to cross-country differences in cultures and societies. Research programs within each theoretical perspective have provided a considerable insight into the origin of democracy by highlighting an important intersection between ecology and society. Nonetheless, several issues remain unsettled. In the following sections, we summarize limitations of the current literature, which were addressed in the present research.

#### Competing hypotheses

The theories described in the previous sections generate mixed predictions regarding the impact of ecological conditions on the development of socio-political freedoms. Broadly speaking, the theories agree that ecological threats are the distal causes of country variations in various domains of human functioning. However, they disagree on the specific roles of such threats in affecting societies. On one hand, researchers argue that pathogens (Hypothesis 1: Thornhill and Fincher, 2014) and natural disasters (Hypothesis 3: Oishi and Graham, 2010) are related to higher collectivism, which has implications for lower levels of socio-political freedoms. On the other hand, some researchers argue that natural disasters increase individualism and democracy (Hypothesis 4: Grossmann and Varnum, 2015). Furthermore, climato-economic theory argues that climatic demands have differential impacts on a society depending on the levels of wealth (Hypothesis 2: Van de Vliert, 2009). These hypotheses raise the possibility that impacts of ecological threats on the levels of socio-political freedoms might be ecology or domain specific.

A more critical limitation is that many of the previous research did not rule out competing hypotheses, although they focused on similar dependent variables (e.g., Murray et al., 2011; Van de Vliert, 2011b; Fincher and Thornhill, 2012a). Diagnostic evidence to support each hypothesis can only be upheld if alternative hypotheses are tested and ruled out in a conservative manner. This issue is particularly pertinent between the present hypotheses, since the ecological factors are expectedly redundant with each other, or to covary highly with third variables such as absolute latitude (Currie and Mace, 2012). Thus, it is worthwhile to test the present hypotheses against each other and isolate the effects of each ecological factor. The present study exactly did so by including all of the proposed predictors in regression models.

#### The controversial role of wealth

There exists a controversy about the role of economic wealth in altering the impact of ecological threats. Climato-economic theory (Van de Vliert, 2009) is the only theory that explicitly incorporates the moderating function of economic wealth on the impacts of ecological threats. However, climato-economic theory also implies that human implications of pathogens and natural disasters should be a function of the severity of ecological threats adjusted by economic resources. For instance, Lin (2015) demonstrated that countries with a higher state capacity (government expenditure divided by GDP) were more successful at reducing the ratio of citizens affected by natural disasters. Democratic regime further moderated this relationship: democratic countries were more effective at using available resources to buffer against the consequence of natural disasters than less democratic countries.

Elaborating on Lin (2015), we tested a new hypothesis that natural disasters and pathogens might have only a weaker relationship with socio-political freedom at higher levels of wealth, whether that relationship is assumed to be positive or negative (Hypothesis 5). Few studies within the above theories have tested this possibility. Fincher and Thornhill (2012b) tested their prediction by including the pathogen–wealth interaction and claimed that the association between pathogen prevalence and individualism remained negative only among *wealthy* countries. This type of relationship was confirmed by Oishi and Komiya (2017) who also showed a negative association between natural disaster risk and individualism exclusively among *wealthy* countries. These findings, however, contradict the prediction of climato-economic theory (Hypothesis 2) and our prediction (Hypothesis 5). Whereas, a compelling explanation for this result by Fincher and Thornhill (2012b) and Oishi and Komiya (2017) remains elusive, the present study sought to clarify this inconsistency by examining the moderating effects of economic wealth on all the target ecological predictors.

#### Problematic operationalization of threat

We argue that some of the conclusions drawn from previous studies are uncertain due to a problematic operationalization of ecological threats. For instance, researchers limited themselves to analyzing disaster frequency and risk as a proxy of disaster threats (Grossmann and Varnum, 2015; Oishi and Komiya, 2017; Santos et al., 2017). However, risk and frequency might be a poor indicator of *threat* without specifying the relative population affected by natural disasters (e.g., India had 14 earthquakes that killed 32,117 people, while the United States had 18 earthquakes that killed 143 people between 1980 and 2002; Kahn, 2005). To address this limitation, we quantified the actual casualties directly caused by natural disasters and built a regression model in which natural disaster threat predicted future development of socio-political freedom.

#### Non-independence of observations in cross-cultural research

Finally, previous research has ignored the possibility that, on average, countries within the same region of the world are more similar to each other than countries from different parts of the world. This phenomenon is known as “Galton's problem” in anthropological literature (e.g., Eff, 2004; Dow, 2007). Because countries are similar to each other on many attributes (e.g., geographical distance, language, history), the assumption of non-independence of observations is hardly ever met in cross-cultural studies using standard OLS regressions (Dow and Eff, 2008). With regard to the present study, the theory on the diffusion of democracy points to the possibility that the state of democracy in a country depends much on political transitions in neighboring countries (Gleditsch and Ward, 2006). From the ecocultural perspective, it is also reasonable to assume that countries located closely to one another face similar circumstances of nature, and these ecological circumstances affect socio-political freedom in similar ways. To ensure the robustness of our results, we followed the advice of Kuppens and Pollet (2014) and added region classification as a random effect to our regression models. This procedure allowed us to obtain proper parameter estimates while explicitly modeling the bias associated with non-independence of countries.

### Overview of the present study

An overarching aim of the present study was to test directly the hypotheses concerning effects of pathogens, climate, and natural disasters in predicting country-variations in socio-political freedom. Accordingly, we conducted a series of country-level analyses that sought to predict variations in three domains of socio-political freedom: political freedom, media freedom, and economic freedom. The advantage of selecting these socio-political indicators includes: (1) the breadth of countries covered; (2) broad domains of socio-political freedom based on multi-items/years methodology to ensure higher reliability; and (3) data from multiple independent organizations to minimize potential biases inherent in the indices of freedom generated by particular organizations (e.g., the effort of influential countries to make their own society look superior in the international comparison). Our analyses were based on a cross-sectional design, which included a series of sequential regressions separately for each measure of socio-political freedom. We first controlled for the effects of covariates. Second, we examined additional variance explained by main effects of the target ecological predictors. Finally, we examined interactions between the ecological predictors and economic wealth. We report parameter estimates of the observed effects by using a linear mixed-effects model.

## Method

### Dependent variables

We obtained data from a variety of different online databases and previously published works. Priority in the selection of dependent variables was given to the following criteria: (1) inclusiveness of countries; (2) widths of years recorded; and (3) multi-item methods. Accordingly, we selected three indicators that represent three major domains of socio-political freedom: political freedom, media freedom, and economic freedom.

#### Political freedom

We selected the unified democracy score (Pemstein et al., 2010) as a proxy for political freedom. The unified democracy score is based on a Bayesian latent variable approach that synthesized ten major indices of democracy from multiple sources in political science literature. The latest dataset contained country scores from 1946 to 2012 for 199 societies (http://www.unified-democracy-scores.org/uds.html). Higher values indicate a higher level of democracy.

#### Media freedom

Freedom House (2017) collects annually survey data regarding freedom of the press from a team of regional experts and scholars across wide geopolitical regions. The respondents in the survey rate the extent to which their press freedom is repressed in their regions from three perspectives: legal environment, political environment, and economic environment. The Freedom House aggregates item scores from these factors to generate press freedom score. We used this index as an indicator of media freedom. The original dataset covered over 200 regions and territories from 2001. We reversed the original press freedom score so that a higher score indicates a higher level of press freedom.

#### Economic freedom

The Heritage Foundation (2017) generates scores of economic freedom by integrating various factors related to economic activities for over 180 countries from 1995. The latest data (2017) included two factors—judicial effectiveness and fiscal health—that were not included prior to 2017. To be consistent with previous research (Thornhill et al., 2009), we omitted these factors and only relied on the composite of original 10 factors. A higher score of economic freedom indicates a higher level of economic freedom.

### Predictor variables

Again, we obtained data from a variety of different online databases and previously published works. As described more fully in the Results section, it was important to obtain data for our predictors pertaining to the time period preceding our dependent variables (freedom).

#### Historical pathogen prevalence

Data for pathogen threat were directly taken from the work of Murray and Schaller (2010). They developed indices of historical pathogen prevalence of pathogen prevalence across geopolitical regions for years since 1944 by consulting epidemiological maps in Rodenwaldt and Bader (1952-1961), Simmons et al. (1944). Broadly similar to Gangestad and Buss (1993), Murray and Schaller (2010) used this information to generate a four-point estimate ranging from 0 *completely absent or never reported* to 3 *present at severe levels or epidemic levels at least once*^3^.

Since historical pathogen prevalence has stronger predictive validity than contemporary pathogen prevalence (Fincher et al., 2008), we selected historical pathogen prevalence as an indicator of pathogen threats. Cronbach's alphas for the nine-items, seven-items, and six-items of pathogen data were as follows: α = 0.84, α = 0.75, and α = 0.70, respectively (Murray and Schaller, 2010). We used the index of historical pathogen prevalence based on seven items (leishmanias, schistosomes, trypanosomes, malaria, typhus, filariae, and dengue) because it has higher reliability than the six-items and covers a larger number of societies than the nine-item index^4^.

#### Climatic demands

We derived the index of climatic demands directly from the work of Van de Vliert (2013b). Climatic demands are the sum of cold demands and heat demands. Cold (heat) demands refers to the sum of the absolute downward (upward) deviations from 22°C for (a) the average lowest temperature in the coldest (hottest) month; and (b) the average highest temperature in the coldest (hottest) month. The original dataset covered 232 regions.

#### Natural disaster casualties

We derived data on natural disaster threat from the work of Lin (2015), who computed the rate of disaster deaths and affected people based on the disaster records provided by Centre for Research on the Epidemiology of Disasters (2017). The number of disaster deaths was based on the number of death tolls directly caused by a disaster. Affected people were defined as (1) people diagnosed with trauma, injuries, or illness requiring medical assistance due to a disaster; (2) people in need of assistance during emergency; (3) people requiring immediate shelter; or (4) people who were displaced or evacuated due to a natural disaster. The type of natural disasters included earthquakes, floods, and storms, as these disaster types are considered most fatal (Centre for Research on the Epidemiology of Disasters, 2017). Then, the annual number of death tolls and affected people were divided by national population and multiplied by 100,000 to be expressed as loss rate per 100,000 citizens per year for available countries. This index was log-transformed for each year and averaged across 1995 and 2009 for 150 countries (Lin, 2015).

In the present study, we call this index natural disaster casualty and use it as a proxy of natural disaster threats. A higher natural disaster casualty score indicates a higher level of physical damage solely caused by major natural disasters. Natural disaster casualty had a high temporal reliability from 1995 to 2009, α = 0.90. We also examined its convergent validity with other disaster-related constructs used in previous research. Natural disaster casualty positively correlated with (a) the mean total occurrence of natural disasters and technological disasters across years to date, *r* = 0.52, *p* < 0.01 (*n* = 150); (b) the mean total occurrence of only natural disasters across years to date, *r* = 0.52, *p* < 0.01 (*n* = 146); (c) the mean total occurrence of only technological disasters across years to date, *r* = 0.42, *p* < 0.01 (*n* = 142); (d) the mean total economic damage caused by natural disasters across years to date, *r* = 0.19, *p* < 0.05 (*n* = 138) (Centre for Research on the Epidemiology of Disasters, 2017). Natural disaster casualty also correlated with the mean risks of natural disasters across 2011, 2012, 2013, and 2016 (Oishi and Komiya, 2017), *r* = 0.56, *p* < 0.01 (*n* = 142)^5^. Therefore, natural disaster casualty holds sufficient reliability and convergent validity, while it uniquely reflects the actual number of people who suffered from natural disasters.

#### Economic wealth

We obtained GDP per capita from The World Bank (2017) as the covariate^6^.

#### Population density

In societies with higher population density, ecological threats might be more consequential (Gelfand et al., 2011). Thus, we included population density as a covariate in our analysis. We obtained population density scores from The World Bank (2017) available for consecutive years for over 200 geopolitical regions.

#### Region classification

We used region classification provided by the United Nations (2017) to model bias associated with non-independence of countries. The United Nations (UN) divide the world region into five major continents solely based on geographical location. These regions are further divided into 22 sub-regions on the basis of similarities in population and demographic characteristics^7^.

## Results

Our sample was restricted to countries for which all predictor variables were available, thus limiting our sample size to 150. Similarly, natural disaster casualty (hereafter disaster) limited the range of years that could be included; with this variable being only available for 1995–2009, only these years for the predictors were included in the present study. The dependent variables were selected during years after 2009 to ensure causal implications (unified democracy score, 2010–2012; press freedom, 2010–2016; economic freedom, 2010–2016, respectively). All available years were averaged to create reliable indicators. Occasionally, missing data had to be imputed based on credible sources as in the case of Taiwan where GDP and population density were imputed based on the available data from 2000 to 2009 in the World Factbook database (Central Intelligence Agency, 2017). Missing values for other countries were imputed with the mean scores of each cluster based on the 22-level region classification to which that country's missing values belonged^8^. This resulted in a final cross-sectional dataset including all variables of our interest. Tables 1, 2 summarize descriptive statistics and correlation matrix of all variables, respectively. GDP and population density were log-transformed prior to the main analyses. We grand-mean centered the target ecological predictors and GDP to examine interactions.

**Table 1:** Descriptive statistics (*N* = 150).

**Variable**	***M***	***SD***	***Mdn***	***Min***	***Max***	***Skewness***	***Kurtosis***
Unified democracy score	0.48	0.82	0.41	−1.41	2.18	0.13	−0.59
Press freedom	59.03	22.14	56.14	9.71	96	−0.16	−0.96
Economic freedom	61.34	10.06	60.71	29.96	89.56	0.03	0.16
Historical pathogen prevalence	0.14	0.64	0.18	−1.18	1.2	−0.16	−0.98
Climatic demands	57.44	23.08	53.5	22	129	0.48	−0.68
Natural disaster casualty	2.91	2.18	2.89	−0.57	8.95	0.35	−0.68
GDP per capita	8547.22	13040.19	2784.33	150.99	69082.21	2.05	3.87
	(7.94)	(1.57)	(7.93)	(5.02)	(11.14)	(0.17)	(−0.98)
Population density	213.72	728.23	72.69	1.58	6373.71	7.62	59.98
	(4.19)	(1.43)	(4.29)	(0.46)	(8.76)	(0.00)	(0.67)

*Numbers in parenthesis represent values for the log-transformed variable*.

**Table 2:** Zero-Order Correlations of Variables in the Present Study (*N* = 150).

	**Variable**	**1**	**2**	**3**	**4**	**5**	**6**	**7**
1.	Unified democracy score							
2.	Press freedom	0.92^**^						
3.	Economic freedom	0.73^**^	0.68^**^					
4.	Historical pathogen prevalence	−0.59^**^	−0.51^**^	−0.48^**^				
5.	Climatic demands	0.28^**^	0.20^*^	0.27^**^	−0.62^**^			
6.	Natural disaster casualty	−0.37^**^	−0.40^**^	−0.40^**^	0.48^**^	−0.35^**^		
7.	GDP per capita	0.70^**^	0.62^**^	0.63^**^	−0.55^**^	0.36^**^	−0.47^**^	
8.	Population density	0.02	0.02	0.34^**^	0.00	−0.17^*^	−0.14	0.19^*^

* *p < 0.05*;

** *p < 0.01*.

Each model was assessed in a blockwise fashion. The first block of each model included the effects of GDP and population density. The second block simultaneously added main effects of historical pathogen prevalence (hereafter, pathogen), climatic demands (hereafter, climate), and disaster. The third block then simultaneously examined interactive effects of the target ecological predictors with GDP. We estimated parameters in the full model by the following equation:

$$\begin{array}{l} Freedom_{ij}\;=\;\beta_{0}\;+\;\beta_{1}GDPCentered_{ij}\\ \;+\;\beta_{2}PopulationDensity_{ij}\\ \;+\;\beta_{3}PathogenCentered_{ij}\;+\;\beta_{4}ClimateCentered_{ij}\;\\ \;+\;\beta_{5}DisasterCentered_{ij}\;\\ \;+\;\beta_{6}(PathogenCentered_{ij})(GDPCentered_{ij})\;\\ \;+\;\beta_{7}(ClimateCentered_{ij})(GDPCentered_{ij})\;\\ \;+\;\beta_{8}(DisasterCentered_{ij})(GDPCentered_{ij})\\ \;+\;e_{ij}\;+\;u_{j} \end{array}$$

where *Freedom*_*ij*_ is the dependent variable (it is separately measured for unified democracy score, press freedom, and economic freedom in the main analyses) for each country *i* of region *j*, *e*_*ij*_ is the between-country residual (level-1) for each country *i* of region *j*, and *u*_*j*_ is the between-region residual (level 2) for region *j*.

### Does multilevel modeling account for non-independence of countries?

We first assessed the need of adding a random intercept by comparing OLS regression (intercept-only) models with random-intercept-only models for each of our three dependent variables (Kuppens and Pollet, 2014). In so doing, we let the intercepts vary by 22 regions for each of the three outcome measures of socio-political freedom. According to the United Nations (2017), sample countries in the present study were clustered within 20 regions (see Table 3). Table 4 summarizes the likelihood ratio tests for each dependent variable to illustrate the degree of non-independence of countries. Significant reductions of Akaike's Information Criteria (AIC) as well as Schwarz's Bayesian Information Criteria (BIC) indicated that adding a random intercept to the intercept-only model significantly improved the model fit for each socio-political freedom. In addition, intraclass correlations (ICC) of each null model suggested that substantial portions of variance in the dependent variables were explained by the differences between regions relative to differences between countries. Notably, for unified democracy score and press freedom, more than half of the total variance was attributable to the region effect. These results clearly demonstrated that countries were inherently interdependent with each other, and multilevel modeling was more appropriate than OLS regression. Accordingly, we report estimates of fixed-effects while allowing the intercepts to vary by 22 regions in the analyses in the subsequent sections using lme4 package in R (Bates, 2010).

**Table 3:** Region classifications by United Nations geographical regions for statistical use.

**5-level**	**22-level**	**Country**
**AFRICA**		
	Northern Africa	Algeria (DZA), Egypt (EGY), Morocco (MAR), Tunisia (TUN)
	Eastern Africa	Burundi (BDI), Comoros (COM), Ethiopia (ETH), Kenya (KEN), Madagascar (MDG), Malawi (MWI), Mauritius (MUS), Mozambique (MOZ), Rwanda (RWA), Tanzania (TZA), Uganda (UGA), Zambia (ZMB), Zimbabwe (ZWE)
	Middle Africa	Angola (AGO), Cameroon (CMR), Chad (TCD), Congo, Dem. Rep. (Kinshasa) (COD), Congo, Rep. (Brazzaville) (COG), Gabon (GAB)
	Southern Africa	Botswana (BWA), Namibia (NAM), South Africa (ZAF), Swaziland (SWZ)
	Western Africa	Benin (BEN), Burkina Faso (BFA), Cabo Verde (CPV), Cote d'Ivoire (CIV), Gambia (GMB), Ghana (GHA), Guinea (GIN), Guinea-Bissau (GNB), Liberia (LBR), Mali (MLI), Mauritania (MRT), Niger (NER), Nigeria (NGA), Senegal (SEN), Sierra Leone (SLE), Togo (TGO)
**AMERICAS**		
	Caribbean	Barbados (BRB), Dominica (DMA), Dominican Republic (DOM), Grenada (GRD), Haiti (HTI), Jamaica (JAM), St. Lucia (LCA), St. Vincent and the Grenadines (VCT), Trinidad and Tobago (TTO)
	Central America	Belize (BLZ), Costa Rica (CRI), El Salvador (SLV), Guatemala (GTM), Honduras (HND), Mexico (MEX), Nicaragua (NIC), Panama (PAN)
	South America	Argentina (ARG), Bolivia (BOL), Brazil (BRA), Chile (CHL), Colombia (COL), Ecuador (ECU), Paraguay (PRY), Peru (PER), Uruguay (URY), Venezuela (VEN)
	Northern America	Canada (CAN), United States of America (USA)
**ASIA**		
	Central Asia	Kazakhstan (KAZ), Kyrgyzstan (KGZ), Turkmenistan (TKM), Uzbekistan (UZB)
	Eastern Asia	China (CHN), Hong Kong (HKG), Japan (JPN), Mongolia (MNG), South Korea (KOR), Taiwan (TWN)^*^
	South-Eastern Asia	Cambodia (KHM), Indonesia (IDN), Lao People's Dem. Rep. (LAO), Malaysia (MYS), Philippines (PHL), Singapore (SGP), Thailand (THA), Viet Nam (VNM)
	Southern Asia	Bangladesh (BGD), Bhutan (BTN), India (IND), Iran, Islamic Rep. (IRN), Maldives (MDV), Nepal (NPL), Pakistan (PAK), Sri Lanka (LKA)
	Western Asia	Armenia (ARM), Azerbaijan (AZE), Cyprus (CYP), Georgia (GEO), Israel (ISR), Jordan (JOR), Lebanon (LBN), Turkey (TUR), Yemen (YEM)
**EUROPE**		
	Eastern Europe	Belarus (BLR), Bulgaria (BGR), Czech Republic (CZE), Hungary (HUN), Republic of Moldova (MDA), Poland (POL), Romania (ROU), Russian Federation (RUS), Slovakia (SVK), Ukraine (UKR)
	Northern Europe	Denmark (DNK), Estonia (EST), Finland (FIN), Iceland (ISL), Ireland (IRL), Latvia (LVA), Lithuania (LTU), Norway (NOR), Sweden (SWE), United Kingdom (GBR)
	Southern Europe	Albania (ALB), Bosnia and Herzegovina (BIH), Croatia (HRV), Greece (GRC), Italy (ITA), Macedonia (MKD), Malta (MLT), Montenegro (MNE), Portugal (PRT), Serbia (SRB), Slovenia (SVN), Spain (ESP)
	Western Europe	Austria (AUT), Belgium (BEL), France (FRA), Germany (DEU), Luxembourg (LUX), Netherlands (NLD)
**OCEANIA**		
	Australia and New Zealand	Australia (AUS), New Zealand (NZL)
	Melanesia	Fiji (FJI), Papua New Guinea (PMG)
	Micronesia	NA
	Polynesia	NA

* *Taiwan was not included in the original UN data*.

**Table 4:** Likelihood ratio tests of non-independence of countries for each dependent variable.

	**Unified democracy score**		**Press freedom**		**Economic freedom**	
	**Intercept only**	**Random intercept**	**Intercept only**	**Random intercept**	**Intercept only**	**Random intercept**
AIC	370	293	1,358	1,295	1,121	1,082
BIC	376	302	1,364	1,304	1,127	1,091
−2 *LL*	366	286	1,354	1,288	1,118	1,076
$\sigma_{e}^{2}$	0.67	0.28	490	234	101	59.1
$\sigma_{u}^{2}$		0.51		299		52.5
ICC		0.65		0.56		0.47
${\chi^{2}}_{\text{change}}$		78.9^***^		65.1^***^		41.3^***^
*df*_change_		1		1		1

*** *p < 0.001*.

### Model 1: unified democracy score

In Model 1, we regressed unified democracy score (hereafter, democracy) on the target ecological predictors and their interactions with economic wealth using a blockwise procedure via maximum likelihood. Table 5 summarizes all coefficients and information statistics. The first block including covariates showed that GDP was the only significant predictor for democracy, indicating that wealthy countries tended to be more democratic (BIC = 240.71; −2 *LL* = 215.66). The second block including main effects of pathogen, climate, and disaster increased the model fit over the first block (BIC = 242.22; −2 *LL* = 202.14), ${\chi^{2}}_{\left(3\right)}$ = 14.10, *p* = 0.003. Consistent with Hypothesis 1, the significant main effect of pathogen suggested that countries with higher pathogen threats were less democratic. The significant main effect of disaster, albeit weaker than pathogen, also suggested that countries with greater natural disaster casualty tended to be more democratic, consistent with Hypothesis 4. The main effect of climate was practically non-existent. In the third block, we added interaction terms, and doing so improved the model fit again over the second block (BIC = 244.76; 2 *LL* = 189.20), ${\chi^{2}}_{\left(3\right)}$ = 12.60, *p* = 0.006.

**Table 5:** Estimated coefficients for unified democracy score (*N* = 150).

**Predictors**	**Block 1**		**Block 2**		**Block 3**	
	**Estimate**	***SE***	**Estimate**	***SE***	**Estimate**	***SE***
Intercept (**β**_**0**_)	0.49^**^	0.15	0.44^**^	0.16	0.39^**^	0.15
ln GDP per capita (**β**_**1**_)	0.38^***^	0.04	0.38^***^	0.04	0.37^***^	0.04
ln Population density (**β**_**2**_)	−0.01	0.03	−0.01	0.03	−0.02	0.03
Historical pathogen prevalence (**β**_**3**_)			−0.36^***^	0.10	−0.34^***^	0.10
Climatic demands (**β**_**4**_)			0.00	0.00	0.00^+^	0.00
Natural disaster casualty (**β**_**5**_)			0.06^*^	0.03	0.05^+^	0.02
Pathogen × GDP (**β**_**6**_)					−0.07	0.06
Climate × GDP (**β**_**7**_)					0.00	0.00
Disaster × GDP (**β**_**8**_)					−0.03^+^	0.02
Country variance ($\sigma_{\text{e}}^{2}$)	0.20		0.19		0.18	
Region variance ($\sigma_{\text{e}}^{2}$)	0.11		0.07		0.04	
ICC	0.34		0.28		0.20	
AIC	225.96		217.84		211.28	

+ *p < 0.10*;

* *p < 0.05*;

** *p < 0.01*;

*** *p < 0.001*.

**Figure 1:**
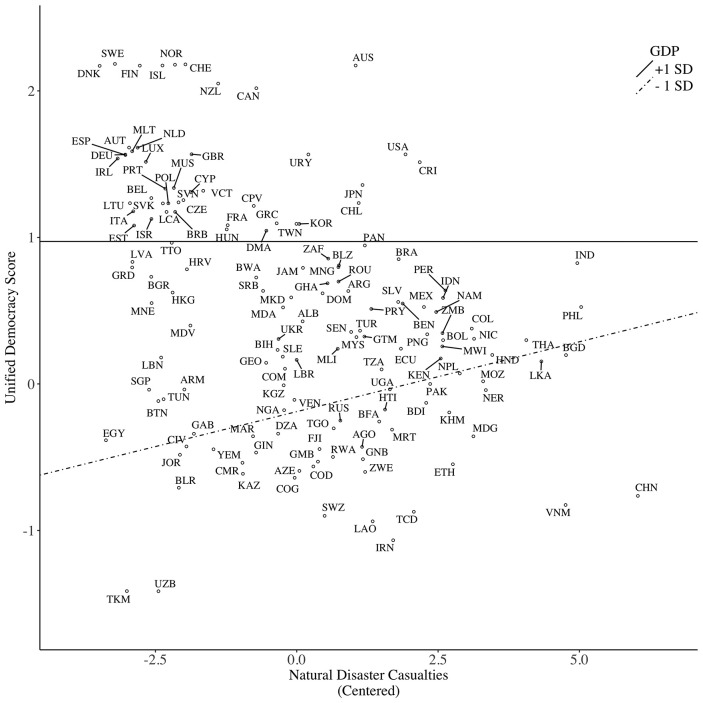
Predicting political freedom (operationalized as Unified Democracy Score) based on casualties caused by natural disasters, moderated by country GDP per capita.

**Figure 2:**
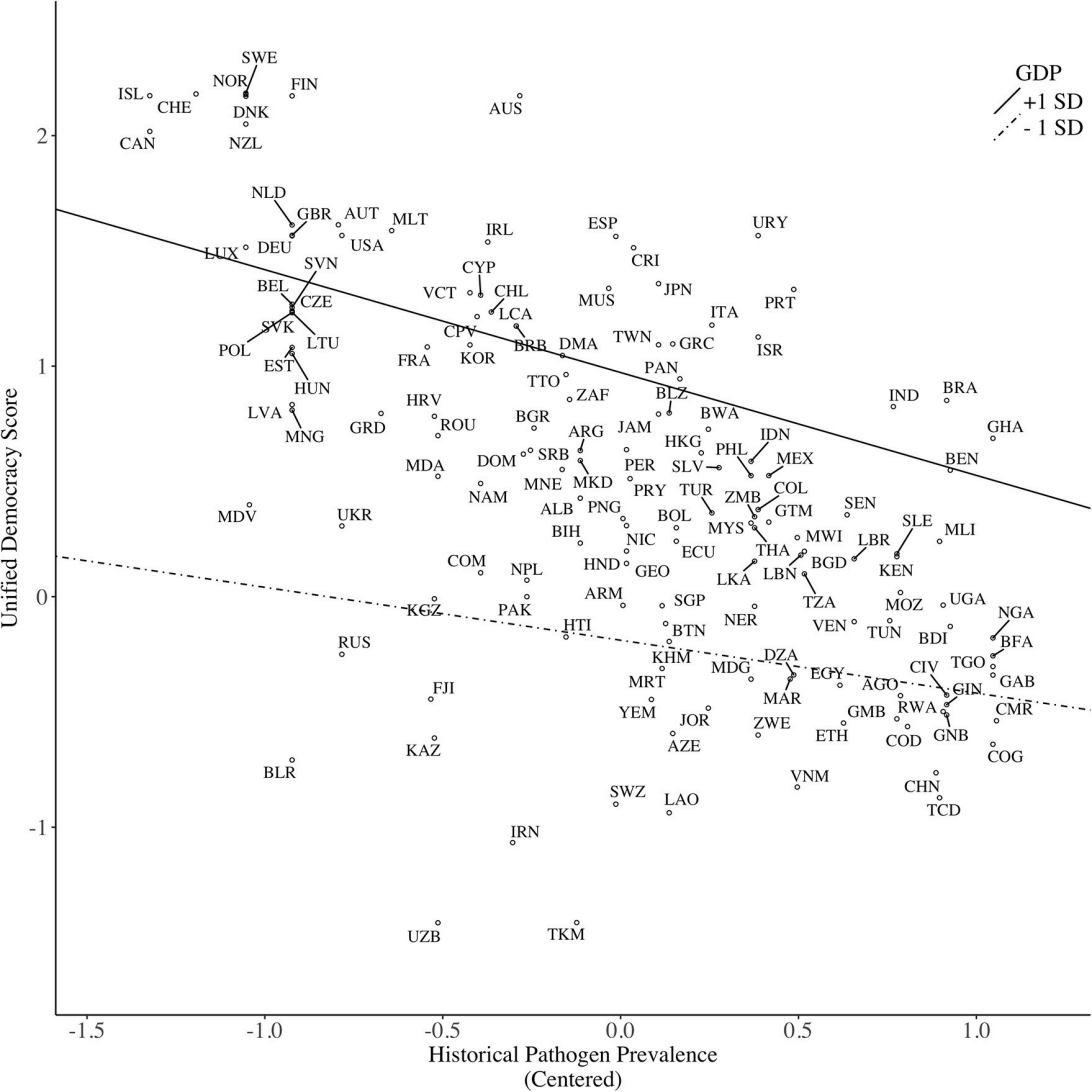
Predicting political freedom (operationalized as Unified Democracy Score) based on historical pathogen prevalence, moderated by country GDP per capita.

The disaster–GDP interaction was marginally significant (*p* = 0.06). A simple slope analysis revealed that disaster had a minor statistical impact on democracy among wealthy (1 *SD* above the mean) countries, *b* = −0.001, *SE* = 0.04, *p* = 0.99; however, disaster was positively related to democracy among poor (1 *SD* below the mean) countries, *b* = 0.10, *SE* = 0.03, *p* = 0.002, indicating that poor countries with higher natural disaster casualty tended to have higher level of democratic freedom (see Figure 1). This pattern confirms the buffering prediction that economic wealth shields against the effect of natural disasters on socio-political freedom (Hypothesis 5). The pathogen–GDP interaction was not reliable (*p* = 0.27)^9^. A *post-hoc* analysis showed that pathogen had a negative effect on democracy among wealthy countries, *b* = −0.45, *SE* = 0.14, *p* < 0.01; however, this effect was only half that size among poor countries, *b* = −0.23, *SE* = 0.14, *p* = 0.10 (see Figure 2). This pattern indicated a small buffering effect of wealth on pathogens among poor countries. Contrary to Hypothesis 2, the climate–GDP interaction had no effect. Adding interaction terms in Block 3 did not alter the main effects of ecological threats.

We also tested the possibility that the observed estimates were inflated by multivariate outliers, using both Cook's D and standardized residuals of greater (smaller) than 3(−3) as criteria. Mongolia, Singapore and Iran emerged as outliers. When these countries were excluded, the model yielded a smaller AIC (and BIC), suggesting improved model fit. But the coefficients remained largely unchanged, with the effects often increasing; therefore, we included these countries in the model reported here, which thus yield conservative estimates. Because similar trends were obtained in subsequent analyses, no further mention will be made on outlier analysis.

### Model 2: press freedom

Following the same procedure as above, Model 2 predicted press freedom using the same predictors (see Table 6 for all coefficients). The first block showed that GDP was the only significant predictor (BIC = 1266.20; −2 *LL* = 1241.12). The second block improved the model fit somewhat over the first block (BIC = 1273.50; −2 *LL* = 1233.42), ${\chi^{2}}_{\left(3\right)}$ = 7.69, *p* = 0.053. Again, consistent with Hypothesis 1, pathogen showed the strongest effect among the target ecological predictors. Unlike Model 1, disaster did not significantly predict press freedom, thus providing no support for Hypothesis 3 and 4. Similarly, climate did not predict press freedom. Finally, the third block improved the model fit over the second block (BIC = 1278.60; −2 *LL* = 1223.48), ${\chi^{2}}_{\left(3\right)}$ = 9.95, *p* = 0.019. Again, contrary to Hypothesis 2, the climate–GDP interaction was very small and not reliable.

**Table 6:** Estimated coefficients for press freedom (*N* = 150).

**Predictors**	**Block 1**		**Block 2**		**Block 3**	
	**Estimate**	***SE***	**Estimate**	***SE***	**Estimate**	***SE***
Intercept (**β**_**0**_)	58.58^***^	4.64	58.65^***^	4.87	58.19^***^	4.68
ln GDP per capita (**β**_**1**_)	8.85^***^	1.12	8.34^***^	1.29	7.93^***^	1.24
ln Population density (**β**_**2**_)	−0.19	0.98	−0.26	1.06	−0.88	1.04
Historical pathogen prevalence (**β**_**3**_)			−8.90^**^	3.23	−8.63^**^	3.11
Climatic demands (**β**_**4**_)			−0.13	0.08	−0.16^*^	0.08
Natural disaster casualty (**β**_**5**_)			0.75	0.81	0.18	0.79
Pathogen × GDP (**β**_**6**_)					−1.47	1.97
Climate × GDP (**β**_**7**_)					0.01	0.05
Disaster × GDP (**β**_**8**_)					−1.22^*^	0.5
Country variance ($\sigma_{\text{e}}^{2}$)	189.12		183.14		176.65	
Region variance ($\sigma_{\text{u}}^{2}$)	92.42		73.41		50.52	
ICC	0.33		0.29		0.22	
AIC	1251.12		1249.43		1245.48	

* *p < 0.05*;

** *p < 0.01*;

*** *p < 0.001*.

**Figure 3:**
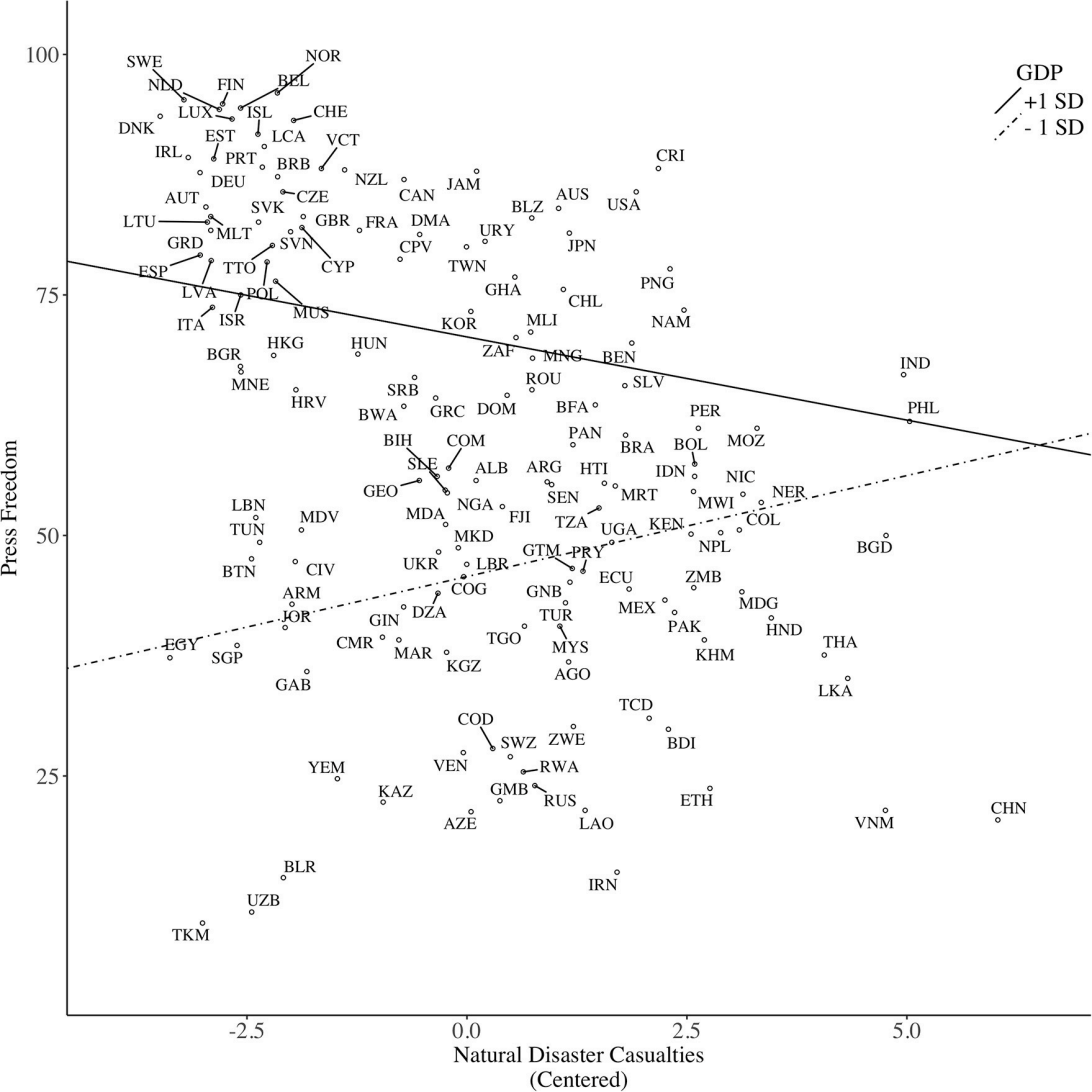
Predicting media freedom (operationalized as Press Freedom) based on casualties caused by natural disasters, moderated by country GDP per capita.

However, the disaster–GDP interaction did surpass significance. Simple slope analysis revealed that disaster was negatively, albeit not significantly, related to press freedom among wealthy countries, *b* = −1.73, *SE* = 1.22, *p* = 0.16, whereas this relationship was positive among poor countries, *b* = 2.10, *SE* = 0.99, *p* = 0.034 (see Figure 3). This pattern is consistent with Model 1 and again supports Hypothesis 5. Yet, the pathogen–GDP interaction was not significant. Block 3 did not alter the significance of the main effect of pathogen; however, the main effect of climate became significant, indicating that countries with greater climatic demands showed lower levels of press freedom.

### Model 3: economic freedom

Model 3 regressed economic freedom on the same predictors as previous models using the same procedure (see Table 7 for all coefficients). In the first block, both GDP and population density were significant predictors of economic freedom (BIC = 1029.10; −2 *LL* = 1004.10), indicating that in addition to wealth, countries with higher population density had more freedom in economic infrastructures. Unlike the previous models, the second block worsened the model fit (BIC = 1042.50; −2 *LL* = 1002.36), ${\chi^{2}}_{\left(3\right)}$ = 1.72, *p* = 0.63, as none of the main effects were related to economic freedom. This null result is inconsistent with Hypothesis 1, 3, and 4. The third block also did not improve the mode fit, though the deviance decreased (BIC = 1052.70; −2 *LL* = 997.57), ${\chi^{2}}_{\left(3\right)}$ = 4.80, *p* = 0.19.

**Table 7:** Estimated coefficients for economic freedom (*N* = *150*).

**Predictors**	**Block 1**		**Block 2**		**Block 3**	
	**Estimate**	***SE***	**Estimate**	***SE***	**Estimate**	***SE***
Intercept (**β**_**0**_)	57.33^**^	1.91	56.49^**^	2.07	56.32^**^	2.07
ln GDP per capita (**β**_**1**_)	4.50^**^	0.44	4.26^**^	0.57	4.18^**^	0.55
ln Population density (**β**_**2**_)	0.96^*^	0.43	1.16^*^	0.47	1.07^*^	0.47
Historical pathogen prevalence (**β**_**3**_)			−1.24	1.44	−1.39	1.41
Climatic demands (**β**_**4**_)			0.02	0.04	0.01	0.04
Natural disaster casualty (**β**_**5**_)			0.14	0.36	0.17	0.35
Pathogen × GDP (**β**_**6**_)					−1.95^*^	0.92
Climate × GDP (**β**_**7**_)					−0.04^+^	0.03
Disaster × GDP (**β**_**8**_)					0.02	0.23
Country variance ($\sigma_{\text{e}}^{2}$)	43.54		42.69		42.15	
Region variance ($\sigma_{\text{u}}^{2}$)	5.28		5.91		4.20	
ICC	0.11		0.12		0.09	
AIC	1014.09		1018.37		1019.57	

+ *p < 0.10*;

* *p < 0.05*;

** *p < 0.01*;

^***^ p < 0.001.

**Figure 4:**
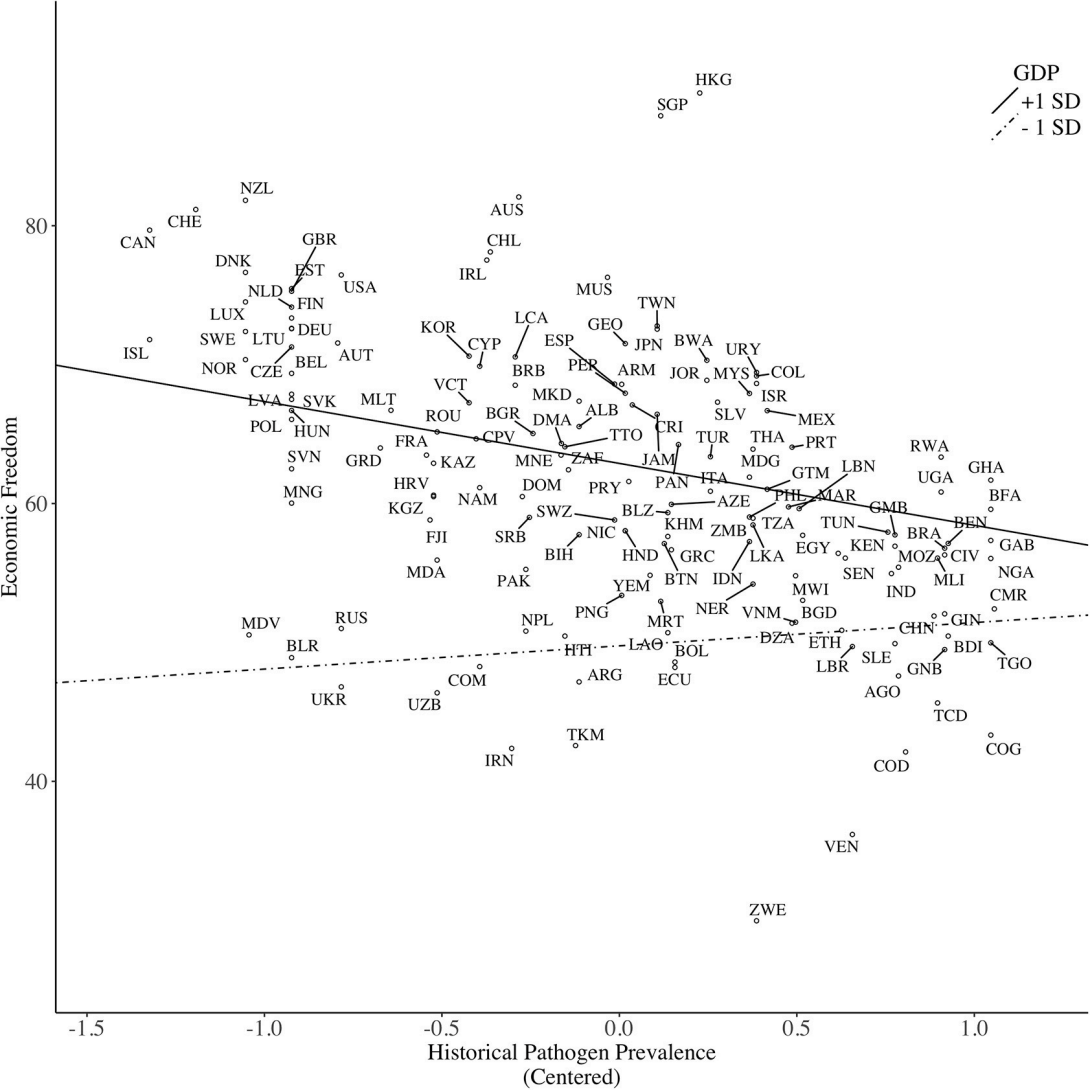
Predicting economic freedom based on historical pathogen prevalence, moderated by country GDP per capita.

**Figure 5:**
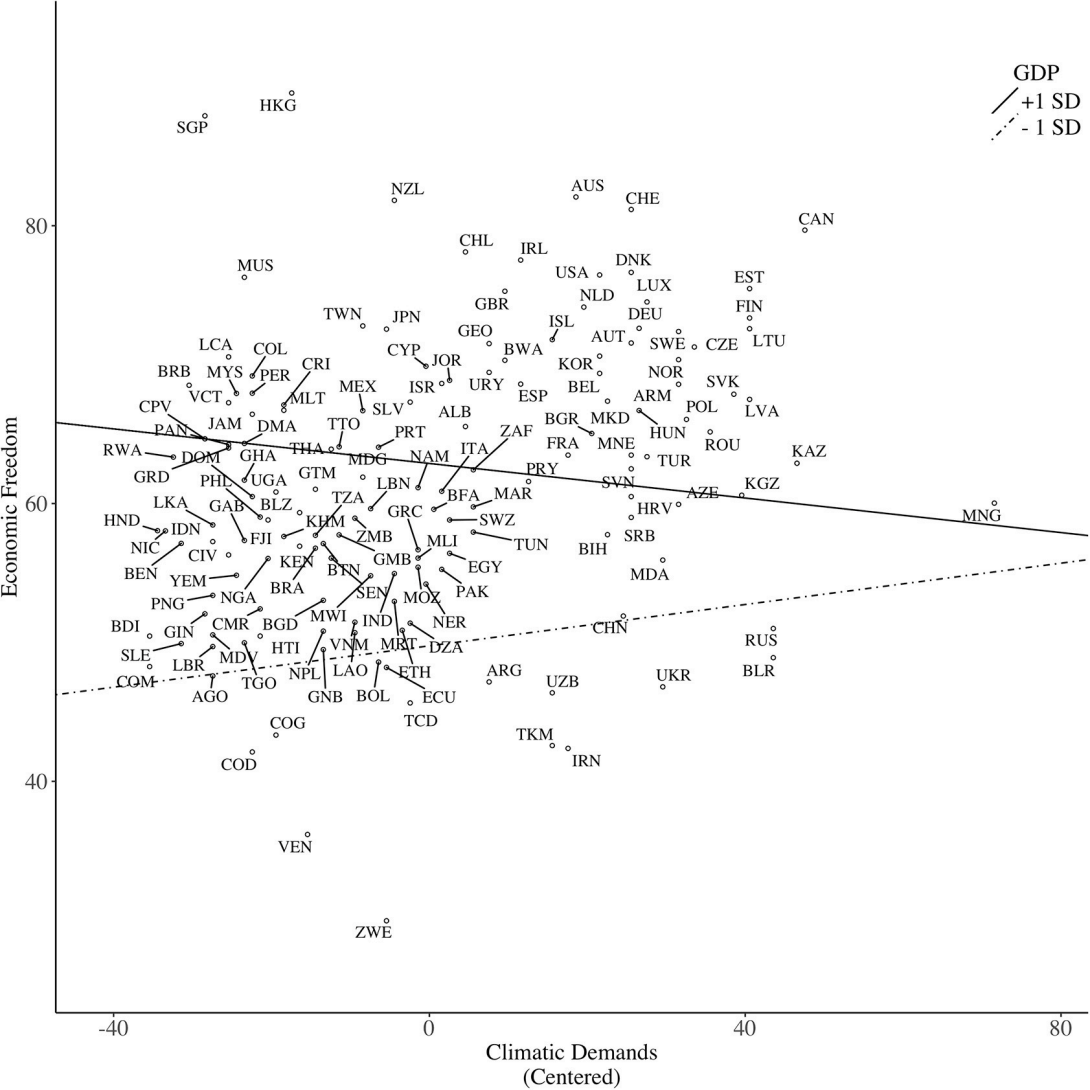
Predicting economic freedom based on climatic demands, moderated by country GDP per capita.

Nonetheless, consistent with Model 1, the pathogen–GDP interaction emerged, with there being a negative effect of pathogen among wealthy countries, *b* = −4.46, *SE* = 2.07, *p* = 0.031, but not among poor countries, *b* = 1.68, *SE* = 1.97, *p* = 0.394, with the slope being slightly positive (see Figure 4). This pattern contradicts Hypothesis 1, and the buffering prediction, Hypothesis 5. Furthermore, the climate–GDP interaction was marginally significant (*p* = 0.092): among wealthy countries, the slope was slightly negative, *b* = −0.06, *SE* = 0.06, *p* = 0.278, indicating that wealthy countries with harsher climate had lower levels of economic freedom. On the other hand, the slope was slightly positive among poor countries, *b* = 0.07, *SE* = 0.05, *p* = 0.14 (see Figure 5). This interaction pattern, however, is incompatible with Hypothesis 2, which predicts that climate is positively associated with socio-political freedom among wealthy countries but negatively among poor countries. Contrary to Model 1 and 2, the disaster–GDP interaction was not significant.

### Testing for robustness

To ensure that the present findings are robust, we repeated our analyses by using complete cases only; in other words, we did not impute data but employed listwise deletion. Analyses only confirmed those reported here^10^. Likewise, rather than adding blocks of variables, we used a criterion-guided deletion process based on which effects whose significance levels did not surpass at least α = 0.10 were removed from the model, unless they were involved in a higher-order interaction, which did surpass this threshold. Again, results were very similar^11^.

## Discussion

We tested five hypotheses concerning the roles of pathogens, climate, natural disasters, and their interactions with economic wealth in predicting country variations in the level of socio-political freedom. Overall, the results did not straightforwardly favor one theory over another. Our results mostly confirmed Hypothesis 1 such that pathogen was the strongest ecological predictor, and countries with higher pathogen threat were less democratic and more likely to suppress press freedom than countries with lower pathogen threats. The hypothesized climate–wealth interaction was negligible across all analyses, providing no support for Hypothesis 2. Economic wealth moderated the impact of natural disaster on democracy and press freedom: poor countries with higher natural disaster threats had a higher level of democracy and press freedom, whereas that relationship was in the weaker (or opposite) direction among wealthy countries; thus supporting Hypothesis 5 but not Hypothesis 3 and 4. Furthermore, we revealed that variance due to clusters of countries accounted for the substantial portion of the variance in socio-political freedoms, thus justifying the use of multilevel modeling. In the following section, we evaluate each theory in light of the observed findings. We next focus on methodological and theoretical implications of the present study.

### Pathogen-stress theory (hypothesis 1)

In the present study, the effect of pathogen remained robust in predicting democracy and press freedom. Nonetheless, Model 1 and 3 revealed interaction effects: the negative effect of pathogen was only observed among wealthy countries but not poor countries. These findings are incompatible with the central logic of pathogen-stress theory that pathogens hinder societies from becoming more individualistic, democratic, and affluent (Thornhill et al., 2009; Murray, 2014). Based on pathogen-stress theory, there is no reason to expect that poor countries would not suffer from pathogens in the development of economic freedom. We speculate that institutions in poor countries with greater pathogen risks may invest more on economic development, believing that the pursuit of economic freedom will result in greater technological and medical innovations (e.g., Arrow, 1962; Miller et al., 2015). If successful, this should reduce susceptibility to pathogen risk. Future research should examine this notion with additional data.

### Climato-economic theory (hypothesis 2)

Remarkably, the results across analyses yielded no support for climato-economic theory. Only a weak interaction between climate and GDP was observed in predicting economic freedom, yet its implication was the opposite of the core prediction of climato-economic theory. It is noteworthy that the null finding in Model 2 runs counter to a previous study linking climate–wealth interaction with the level of press repression as demonstrated by Van de Vliert (2011a); however, in this particular study, pathogens and natural disasters were not examined. To our knowledge, no previous studies by climato-economic theory examined natural disasters as a rival predictor, though a few did rule out pathogen prevalence as a rival explanation (Fischer and Van de Vliert, 2011; Van de Vliert, 2013b). Equally, we know of no studies that examined climate–wealth interaction on economic freedom, for which our data did not provide support. As a follow-up analysis, we indeed replicated the original climate–wealth interaction in the model predicting democracy and press freedom, but only when climate and GDP were the only predictors^12^. That is, either one of pathogen, disaster, and the disaster–GDP interaction was strong enough to invalidate the climate–wealth interaction. The null result may conform with the fact that researchers have challenged climato-economic theory and questioned the validity of climatic demands (e.g., see Fincher and Thornhill, 2012b for a critique).

In our view, it is possible that climato-economic theory is correct, but that pathogens merely “transmit” the effects of climate onto freedom. Warmer climates, especially characterized by higher range of precipitation, give rise to more virulent infectious diseases in humans (Guernier et al., 2004). Also, historically the relative affluence of a society may have been relatively constant, meaning that even historically some societies may have been more (or less) successful at combatting the spread of infectious diseases. In other words, at least conceptually a mediational model is possible: climate (or capacity to guard against negative implications of climate) → pathogens → freedom. Whereas it is possible, at present there is no conclusive evidence that would allow one to distinguish between this mediational model, and a model in which climato-economic theory is merely insufficient in explaining variations in socio-political freedom.

### The role of natural disasters (hypothesis 3 and 4)

Across all analyses, we found no strong evidence for Oishi and Komiya (2017), whose approach predicts a linear negative association between natural disasters and socio-political freedom. On the other hand, the observed positive main effect of disaster on press freedom, albeit marginal, seems to support the social orientation approach (Varnum et al., 2010). However, the social orientation approach cannot account for the observation that the positive effect of disaster was only obtained among poor countries, but not among richer countries. The interaction is instead in line with the buffering prediction (Hypothesis 5). Nonetheless, no theories tested in the present study can account for the positive association between disaster and socio-political freedom among poor countries.

We submit that the observed patterns may coincide with the political agency model (Besley and Burgess, 2002), which argues that media plays a central role in enhancing government responsiveness to vulnerable citizens in disaster-prone areas. The model is based on the idea that, as citizens are more likely to vote for politicians who are attentive to local needs, effective use of media enhances election chances as a means of communicating politicians' responsiveness with citizens. As a result, effective media freedom might be especially pertinent in poor countries, in which citizens are more vulnerable due to limited resources. In contrast, in wealthy countries the media play a lesser role in enhancing politicians' responsiveness, simply because citizens have access to greater resources and suffer less from natural disasters. In other words, especially in poor countries maximizing socio-political freedom might serve as an effective strategy to increase chances of collective survival.

### The role of economic wealth (hypothesis 5)

We predicted that, in wealthy countries, ecological threats have less influences on socio-political freedom because available economic resources alleviate the impact of threats. Models 1 and 2 confirmed this prediction and showed that natural disaster casualty was unrelated to democracy and only weakly (negatively but not significantly) related to press freedom among wealthy countries. In contrast, Model 3 did not support this prediction, as the buffering effect of wealth on pathogen was observed among poor countries. Despite the lack of economic resources, economic freedom among poor countries might be relatively more affected by pathogen risks than natural disasters, with pathogens driving higher economic freedom. Future research may investigate the ecology-specific role of economic wealth in the development of socio-political freedom.

### Methodological implications

The present study offers important methodological implications. First, our research demonstrated a need to focus on multiple indicators of the same construct. Specifically, we focused on the three socio-political indicators of freedom, on which a large number of countries were measured. This strength allowed us to test each theory more comprehensively and detect the ecology- and domain-specific roles of economic wealth, relatively unexamined in previous research. Many country-level indicators of cultural constructs such as cultural dimensions (Hofstede, 2001; House et al., 2004), values (Schwartz, 1992), and personality traits (Allik et al., 2017) tend to lack data on poor countries. Equally, country-level research on the interplay between ecology and culture have exclusively relied on these variables as the dependent measurements. This difference in sample size might explain the gap between our findings and previous findings regarding the role of economic wealth. We await future research that collects data from more poor countries or small-scale societies to illuminate the potential moderating role of wealth.

Second, we have shown that the number of natural disaster casualty emerged as an important predictor of freedom. In contrast to other disaster-related measures used before, such as disaster frequency or natural disaster risk, or even climatic demands, natural disaster casualty taps the actual implications of disaster more accurately. Indeed, juxtaposition of natural disaster casualty with these previous measures reveals that only natural disaster casualty assessed the actual impact of disaster on society, whereas threat was merely *assumed* assessing disaster frequency/risk or climatic demands^13^. The central assumption in the literature, foremost by Berry (2002), is that ecological threat shapes culture and society. We urge that the actual detrimental impact of ecological circumstances on society must be documented, if only to guard against the possibility that the same kind of natural disaster may affect many people in one situation, but only very few in another. In other words, it cannot be assumed that there is an immediate link between an event and its implications for society. Yet, this appears to be a tacit assumption of climato-economic theory, which focused on absolute deviations from comfortable temperature, with greater deviations posing greater levels of survival threats. Again, we argue that, to test this assumption, an appropriate approach is to quantify physical implications associated with threats directly. The present findings support our position: natural disaster casualties refer to the actual number of people who were physically harmed by natural disasters. In a similar vein, historical pathogen prevalence used in the present study might capture human implications, as it is based on reported incidence of infectious diseases (Murray and Schaller, 2010). As a result, these predictors appeared to be more significant than climatic demands. We therefore recommend that cross-cultural research on climate rethink the validly of measurement. For instance, death rate due to heat was positively associated with cultural tightness in U.S. states (Harrington and Gelfand, 2014). Alternately, climate might impose threats on humans indirectly by triggering interpersonal conflicts (Hsiang et al., 2013). Climatic demands could still be a valuable predictor in the mediational model described earlier, but this possibility needs to be tested in future research.

Our study also demonstrated the merit of modeling the interdependence of countries. This result might be unsurprising, given that there are many ways in which countries have become interdependent with each other (see Pollet et al., 2014 for more methodological insights on this issue). Although the present study treated this regional clustering as a statistical nuisance for estimating parameters of ecological predictors, the observed results might stimulate explorations for factors accounting for the interdependence of societal freedom. While the region variable used in the present study was only a crude estimate of clusters of countries, future research may explicitly measure the complex integration process. Historical events, migration, international relations, or other complex acculturation processes have led to the similarities between countries that exist today. For example, Gleditsch and Ward (2006) found that the proportion of democratic states within a 500 km of a country predicted propensity of democratic transitions. More sophisticated methods to account for interdependence of countries might involve modeling autocorrelation effects on various ecological variables across regions (Dow and Eff, 2008). We emphasize that the issue of interdependence of societal freedom is of great concern in cross-cultural research, given that the study of freedom has been the cornerstone of many influential frameworks in the field (e.g., Hofstede, 2001). Overall, the present study supports the previous argument on this issue (Kuppens and Pollet, 2014) and calls for serious consideration of modeling statistical non-independence of observations in country-level research when geopolitical regions are the unit of analysis at any levels.

We submit that considering regional clustering might also offer a possible path in understanding discrepancies in the results across our three dependent variables. Though our unified democracy, press freedom and economic freedom scores are highly correlated (see Table 2), we observed that the first two variables showed a level of regional interdependence much higher than economic freedom (see ICC, Table 4). We speculate that this pattern indicates that social and political freedom are subject to a slightly different dynamic than economic freedom. Whereas, democracy may spread regionally, as demonstrated by Gleditsch and Ward (2006), the economic freedom may not quite be as regionally “contagious” as political freedom, with different factors predicting economic freedom than political freedom. Though beyond the scope of our present work, future research will need to explore this possibility.

### Theoretical implications

Our results indicated that theories within the ecocultural framework do not always fit the observed data. Perhaps this gap was due to the implicit assumption that cultures and societies are unobstructed, natural products of the given environments. For instance, theorists advocating pathogens have applied the evolutionary assumption that pathogens have been a selective force shaping societies over the course of human history. Accordingly, studies often linked measures of pathogen prevalence with contemporary measurements of societal freedom and seem to justify the causal path (Thornhill et al., 2009). However, there is a gap between the implications of pathogen threats in the evolutionary past and the process by which people manage pathogen risks in the contemporary societies. Today, numerous circumstances such as climate change or medical improvements might influence how pathogens affect human welfare.

The present study shows that economic circumstance might be one such example. In most cases, adding ecology–wealth interactions improved the model fit, implying that ecological influences on contemporary socio-political freedom should be considered in relation to current economic conditions that a society faces. Therefore, we speculate that an integration of evolutionary theorizing and a socioeconomic approach might fit the observed patterns better. Elaborating upon the mediational model described earlier, a theoretical integration of pathogens, climate, natural disasters, and economic factors might also be plausible in accounting for the process that has led to contemporary freedom. It might be desirable to conceptualize these factors as complimentary—rather than competing—with each other and build a statistical model as such.

## Limitations of the present study

The current findings are not without limitations. First, we note that although the selection of socio-political indicators of freedom has some strengths, these indicators are not conceptually identical to the cultural-level indicator of freedom such as individualism, most commonly studied in the cross-cultural research reviewed in the present paper. Thus, any inconsistency between our findings and previous research on Individualism could be due to measurement inconsistency. Second, large-scale indices of country characteristics may reflect certain ideological preferences and political biases. For instance, the economic freedom index used in the present study is generated by the Heritage Foundation, a well-known conservative public policy think tank. Likewise, our press freedom index was obtained from Freedom House, an organization close to the U.S. government. In both instances, it cannot be excluded that the composition of the indices reflects value judgments as to what aspects of freedom are worthy of inclusion. By contrast, the unified democracy score explicitly seeks to avoid too narrow a conception of freedom by including ten different indicators (Pemstein et al., 2010). Whereas we observed a high correlation between press freedom and the unified democracy score (Table 2), the correlation with the economic freedom index is somewhat lower. Though we speculated that economic and political freedoms may not emerge in tandem in the development of a society, it is possible that part of the lower correlation can be explained by different conceptual choices rooted in different ideological preferences. In order for this to be examined empirically, we would need an alternative assessment of economic freedom, which to our knowledge, is not currently available in the literature.

Third, the present research is cross-sectional, thus limiting causal implications of ecological threats. A longitudinal approach is more suitable for identifying societal changes due to ecological conditions than exploring country variations (Oishi et al., 2017; Varnum and Grossmann, 2017). Fourth, we did not scrutinize validity of some of the variables tested in the present study (e.g., historic pathogen prevalence). Instead, our intention was to be in line with the selection of these variables as used in previous research. Indeed, the target variables such as indices of socio-political freedom, GDP per capita, historical pathogen prevalence, and population density are not without weaknesses, and there might be alternative ways to measure concepts and phenomena. However, it was important to use these variables “as they are” to test the target theories against each other, except for the case of disaster risk/frequency, which was undeniably questionable.

A final limitation relates to the use of significance testing in evaluating the hypotheses. Pollet (2013) argues that statistical inferences such as *p*-values do not make sense when the sample closely matches the entire population. In a study concerning a finite population (e.g., provinces, states), as was the case in the present study, the application of finite population correction factor is recommended (Pollet, 2013). We acknowledge the critique, as we have collected samples from nearly 80% of the finite population (193 countries according to the UN). However, this issue was less critical in the present study since its primary purpose was the comparison of parameter estimates between predictors. The finite population correction factor was applicable, but it did not change the interpretations of the results simply because the relative comparison remained identical; therefore, we report uncorrected estimates for ease of interpretation^14^. Nevertheless, readers should interpret the present results with this limitation in mind.

## Conclusion

Why are there such great variations in the level of freedom across countries? We sought to answer this question by testing the major hypotheses from the ecocultural framework. Despite the limitations, we illustrated some ecology- and domain-specific effects of ecological influences and the moderating role of economic wealth in the development of socio-political freedom. The present study informs future cross-cultural research of some important issues to be recognized: measuring broad and multiple domains of the same construct, the breadth of countries, careful conceptualization of chosen variables, and the need to control for statistical non-independence of countries. We believe that handling these issues correctly will facilitate theoretical development and advance understanding of ecological origins of societal freedom. The exploration of the ecology of freedom should continue to be an important scientific endeavor, as the origin of democracy is one of the most important topics to be understood in the social sciences.

## Author contributions

KK collected the data based on publicly available sources and designed the study, with contributions from MK. KK analyzed the data assisted by MK. KK and MK interpreted the results. KK wrote all parts of the paper with contributions from MK.

### Conflict of interest statement

The authors declare that the research was conducted in the absence of any commercial or financial relationships that could be construed as a potential conflict of interest.

## Footnotes

^1^Ecocultural studies often treat social systems interchangeably with cultural systems. A social system refers to contexts, institutions, and structures, whereas a cultural system refers to a set of values, beliefs, attitudes, norms, and ways of behaving. Different types of measurements are available for each concept at a country-level analysis. Although these concepts are different, the specificity of the causal link between them is not assumed in this paper, as both systems are likely to share common elements characterized by the same ecological conditions. Interested readers might refer to Berry (2002) for details.

^2^Note that the evidence supports that individualism (collectivism) cause individuals to exhibit a more analytical (holistic) cognitive styles. Whereas the authors use this evidence to support the reverse, namely, that an analytical (holistic) cognitive style renders individuals more likely to be individualists (collectivists). We know of no direct evidence pertaining to cultural differences that would support this latter causal claim.

^3^One of our reviewers highlighted that the scientific basis of these estimates remains vague, and procedures for creating the original estimates of the sources used by Murray and Schaller (2010) are not transparent. We submit that the estimates provided by Murray and Schaller be regarded as ‘educated guesses,’ which are included here primarily because we seek to be consistent with prior work on pathogen prevalence as cultural antecedent.

^4^Some countries in the original dataset were measured on six items (Azores, Christmas Island, Maldives, Norfolk Island, St. Helena, and Wake Island).

^5^Data are available at https://en.wikipedia.org/wiki/List_of_countries_by_natural_disaster_risk.

^6^The Gini index was initially considered for inclusion into the model because income inequality could be contributing to lower levels of freedom (Thornhill et al., 2009). However, we excluded it from the present analysis due to high multicollinearity (VIF > 14 for the three models). See supplemental material.

^7^In addition, absolute latitude, gathered from the World Factbook in Central Intelligence Agency (Central Intelligence Agency, 2017), was considered in our models. However, it was not included in the present analysis due to high multicollinearity (VIF > 6 for all three models). See supplemental material.

^8^Imputed values are summarized in Supplemental Material.

^9^Note that this interaction became significant at *p* < 0.05 when we applied the finite population correction factor (Pollet, 2013).

^10^The results remained identical compare to the dataset to which we had employed multiple imputation.

^11^See R syntax and additional details in Supplementary Material.

^12^Note that multilevel modeling correcting for the interdependence of countries weakened these associations. See Supplemental Material.

^13^Note that our measure of natural disaster threat excludes human-made disasters—fires, chemical spills, and transportation accidents—as used by earlier researchers (Grossmann and Varnum, 2015; Santos et al., 2017). We argue that inclusion of human-made disasters in the index of natural disaster threats is not recommended for two reasons. First, because individualism is associated with technological and economic development (Hofstede, 2001), human-made disasters could be a consequence of increased individualism, not one of its antecedents. Second, a close inspection of the data provided by Centre for Research on the Epidemiology of Disasters (2017) indicates that the data on affected people for each country are sparse in any given years, and the impact of human-made disasters on citizens per 100,000 seems trivial. This means that human-made disasters might impose little or no societal implications. To test these assumptions, we examined whether human-made disaster would generate the same effects as natural disasters. We did so by replacing natural disaster casualty with human-made disaster in the all models predicting freedom. Throughout analyses, human-made disaster had no effect on freedom, while estimates of all the other predictors remained identical to those reported in the present paper. This pattern also held consistent when we used a complete dataset without imputations. These results suggest that human-made disasters should not be confused with natural disaster threats. Results are provided in Supplemental Material.

^14^Note that the finite population correction reduced standard errors, which rendered most of the observed trend-level effects (below *p* < 0.10) statistically significant. For example, the pathogen–GDP interaction on democracy in Model 1 became significant at *p* < 0.05.
