# Supplementary material for: Ecology of Freedom: Competitive Tests of the Role of Pathogens, Climate, and Natural Disasters in the Development of Socio-Political Freedom
Source: Front Psychol. 2018 Jun 12;9:954. doi: 10.3389/fpsyg.2018.00954 (PMC6005876; doi:10.3389/fpsyg.2018.00954)
Supplement: Supplementary file 1 [file Data_Sheet_1.ZIP › Supplemental material Tables 04262018.pdf]

TABLE OF CONTENTS

Table 1: Summary of Imputed Values

Table 2: Models Predicting Unified Democracy Score: Data w/o Outliers, Listwise Data, Data with Multiple Imputation, and Backward Procedure

Table 3: Models Predicting Press Freedom: Data w/o Outliers, Listwise Data, Data with Multiple Imputation, and Backward Procedure

Table 4: Models Predicting Economic Freedom: Data w/o Outliers, Listwise Data, Data with Multiple Imputation, and Backward Procedure

Table 5: OLS Models Predicting Unified Democracy Score, Press Freedom, and Economic Freedom

Table 6: Estimated Coefficients for Climato-Economic Models (OLS): Unified Democracy Score, Press Freedom, and Economic Freedom

Table 7: Estimated Coefficients for Climato-Economic Models (Multilevel): Unified Democracy Score, Press Freedom, and Economic Freedom

Table 8: Replications for All Multilevel Models Based on Human-Made Disaster with Imputed Data

Table 9: Replications for All Multilevel Models Based on Maddison's Historical GDP data in 1950

Table 10: Replications for All Multilevel Models Based on Maddison's Historical GDP data in 1929

Table 1

Summary of Imputed Values

Variable	Country	Group-Mean Imputation (by Region)	MICE (m = 5)
Unified Democracy Score			
	Hong Kong	0.62	1.05
Economic Freedom			
	Grenada	64	78.1
Historical Pathogen Prevalence			
	Montenegro	-0.02	-0.78
Gini coefficient			
	Bhutan	0.56	0.63
	Grenada	0.67	0.62
	Taiwan	0.75	0.79
	Turkmenistan	0.65	0.68
Human-made disasters			
	Barbados	1.34	2.29
	Bhutan	-0.33	0.75
	Botswana	0.56	-1.12

Grenada	1.34	2.51
Iceland	0.78	-0.55
Jamaica	1.34	-0.07
Latvia	0.78	-0.19
Mauritius	-0.26	0.11
Montenegro	-0.09	0.67
Republic of Moldova	-0.92	-0.14
Saint Lucia	1.34	0.75
St. Vincent and the Grenadines	1.34	2.76
Taiwan	-0.52	-1.58

Table 2

Models Predicting Unified Democracy Score: Data w/o Outliers, Listwise Data, Data with Multiple Imputation, and Backward Procedure

Predictors	w/o Outliers		Listwise		MICE		Backward	
	b	*SE*	*b*	*SE*	*b*	*SE*	*b*	*SE*
Intercept (β_0)	0.26^{+}	0.14	0.41**	0.15	0.39*	0.15	0.32***	0.07
ln GDP per capita (β_1)	0.41***	0.04	0.36***	0.04	0.37***	0.04	0.36***	0.04
ln Population density (β_2)	0.01	0.03	-0.02	0.03	-0.02	0.03		
Historical pathogen prevalence (β_3)	-0.28**	0.09	-0.33**	0.10	-0.33**	0.10	-0.35***	0.10
Climatic demands (β_4)	-0.01*	0.00	-0.01*	0.00	-0.01*	0.00	0.00^{+}	0.00
Natural disaster casualty (β_5)	0.05*	0.02	0.04	0.03	0.05^{+}	0.03	0.05*	0.02
Pathogen x GDP (β_6)	-0.08	0.06	-0.07	0.06	-0.06	0.06	-0.11*	0.05
Climate x GDP (β_7)	0.00	0.00	0.00	0.00	0.00	0.00		
Disaster x GDP (β_8)	-0.02^{+}	0.01	-0.03*	0.02	-0.03*	0.02	-0.03^{+}	0.02
Country variance (σ_e^2)	0.150		0.183		0.183		0.183	
Region variance (σ_u^2)	0.046		0.035		0.044		0.045	
ICC	0.236		0.162		0.195		0.196	
AIC	182.727		202.609		212.281		208.675	
N	147		144		150		150	

Notes. $^{+}p < .10$; $*p < .05$; $**p < .01$; $***p < .001$

Table 3

Models Predicting Press Freedom: Data w/o Outliers, Listwise Data, Data with Multiple Imputation, and Backward Procedure

Predictors	w/o Outliers		Listwise		MICE		Backward	
	b	*SE*	*b*	*SE*	*b*	*SE*	*b*	*SE*
Intercept (β_0)	54.92***	4.57	58.49***	4.79	58.71***	4.68	55.48***	2.26
ln GDP per capita (β_1)	9.06***	1.20	7.75***	1.25	7.84***	1.24	7.79***	1.24
ln Population density (β_2)	-0.01	1.02	-0.97	1.08	-1.00	1.04		
Historical pathogen prevalence (β_3)	-7.32*	2.97	-8.46**	3.13	-8.83**	3.10	-8.93**	3.13
Climatic demands (β_4)	-0.19*	0.08	-0.18*	0.08	-0.18*	0.08	-0.13$^+$	0.08
Natural disaster casualty (β_5)	0.18	0.75	0.01	0.83	0.16	0.80	0.32	0.79
Pathogen x GDP (β_6)	-1.91	1.87	-1.64	1.98	-1.50	1.98		
Climate x GDP (β_7)	0.01	0.05	0.01	0.05	0.01	0.05		
Disaster x GDP (β_8)	-1.12*	0.48	-1.26*	0.51	-1.23*	0.50	-1.31**	0.46
Country variance (σ_e^2)	161.050		181.005		178.604		176.049	
Region variance (σ_u^2)	38.241		40.813		50.242		61.426	
ICC	0.192		0.184		0.220		0.259	
AIC	1205.279		1197.439		1246.918		1241.586	
N	147		144		150		150	

Notes. $^+p < .10$; $*p < .05$; $**p < .01$; $***p < .001$

Table 4

Models Predicting Economic Freedom: Data w/o Outliers, Listwise Data, Data with Multiple Imputation, and Backward Procedure

Predictors	w/o Outliers		Listwise		MICE		Backward	
	b	*SE*	*b*	*SE*	*b*	*SE*	*b*	*SE*
Intercept (β_0)	57.73***	1.91	57.20***	2.12	56.52***	2.10	56.44***	2.05
ln GDP per capita (β_1)	4.04***	0.50	4.00***	0.56	4.12***	0.56	4.08***	0.51
ln Population density (β_2)	0.88*	0.43	0.83^{+}	0.48	1.06*	0.48	1.04*	0.45
Historical pathogen prevalence (β_3)	-1.67	1.31	-1.66	1.41	-1.85	1.42	-1.29	1.40
Climatic demands (β_4)	0.00	0.03	0.01	0.04	-0.01	0.04	0.01	0.04
Natural disaster casualty (β_5)	-0.02	0.32	0.17	0.37	0.10	0.36		
Pathogen x GDP (β_6)	-1.85*	0.80	-2.09*	0.91	-2.06*	0.93	-1.90*	0.86
Climate x GDP (β_7)	-0.05*	0.02	-0.04^{+}	0.03	-0.05^{+}	0.03	-0.04^{+}	0.03
Disaster x GDP (β_8)	0.12	0.21	0.08	0.23	0.03	0.2		
Country variance (σ_e^2)	29.445		40.376		43.209		42.013	
Region variance (σ_u^2)	7.406		4.594		4.519		4.563	
ICC	0.201		0.102		0.095		0.098	
AIC	949.918		974.610		1023.683		1015.786	
N	146		144		150		150	

Notes. $^{+}p < .10$; $*p < .05$; $**p < .01$; $***p < .001$

Table 5
OLS Models Predicting Unified Democracy Score, Press Freedom, and Economic Freedom (N = 150)

Predictors	Unified Democracy		Press Freedom		Economic Freedom	
	b	*SE*	*b*	*SE*	*b*	*SE*
Intercept (β_0)	0.43**	0.14	58.63***	4.42	56.41***	2.01
ln GDP per capita (β_1)	0.37***	0.04	7.84***	1.15	4.18***	0.52
ln Population density (β_2)	-0.02	0.03	-0.91	1.02	1.01 *	0.46
Historical pathogen prevalence (β_3)	-0.33***	0.10	-8.87**	3.04	-1.24	1.38
Climatic demands (β_4)	-0.01*	0.00	-0.24**	0.08	0.00	0.03
Natural disaster casualty (β_5)	0.04$^+$	0.02	-0.28	0.74	0.22	0.34
Pathogen x GDP (β_6)	-0.10	0.06	-3.64$^+$	2.03	-2.29*	0.92
Climate x GDP (β_7)	0.00	0.00	0.00	0.06	-0.05$^+$	0.03
Disaster x GDP (β_8)	-0.03	0.02	-0.98$^+$	0.52	0.05	0.23
R^2	.67		.54		.54	

Notes. $^+p < .10$; $*p < .05$; $**p < .01$; $***p < .001$

Table 6

Estimated Coefficients for Climato-Economic Models (OLS): Unified Democracy Score, Press Freedom, and Economic Freedom

Predictors	Unified Democracy Score		Press Freedom		Economic Freedom	
	b	*SE*	*b*	*SE*	*b*	*SE*
Intercept (β_0)	0.40***	0.05	57.08***	1.45	61.27***	0.65
ln GDP per capita (β_1)	0.40***	0.03	9.73***	0.92	4.59***	0.41
Climatic demands (β_4)	0.00	0.00	-0.10	0.06	-0.01	0.03
Climate x GDP (β_7)	0.01***	0.00	0.13**	0.04	0.00	0.02
R^2	.618		.480		.501	
N	150		150		150	

Notes. ^{+}p < .10; *p < .05; **p < .01; ***p < .001

Table 7

Estimated Coefficients for Climato-Economic Models (Multilevel): Unified Democracy Score, Press Freedom, and Economic Freedom

Predictors	Unified Democracy Score		Press Freedom		Economic Freedom	
	b	*SE*	*b*	*SE*	*b*	*SE*
Intercept (β_0)	0.38***	0.08	56.92***	2.38	61.37***	0.84
ln GDP per capita (β_1)	0.38***	0.03	8.96***	1.07	4.68***	0.46
Climatic demands (β_4)	0.00	0.00	-0.03	0.07	0.00	0.03
Climate x GDP (β_7)	0.00**	0.00	0.07	0.04	-0.01	0.02
Country variance (σ_e^2)	0.200		190.655		44.815	
Region variance (σ_u^2)	0.073		72.935		5.780	
ICC	0.266		0.277		0.114	
AIC	221.404		1250.808		1020.981	
N	150		150		150	

Notes. ^+p < .10; **p* < .05; ***p* < .01; ****p* < .001

Table 8

Replications for All Multilevel Models Based on Human-Made Disaster with Imputed Data (N = 150)

Predictors	Unified Democracy Score		Press Freedom		Economic Freedom	
	b	*SE*	*b*	*SE*	*b*	*SE*
Intercept (β_0)	0.39*	0.15	57.58***	4.70	56.59***	2.03
ln GDP per capita (β_1)	0.34***	0.04	7.82***	1.15	4.09***	0.51
ln Population density (β_2)	-0.01	0.03	-0.43	1.03	1.00*	0.45
Historical pathogen prevalence (β_3)	-0.31**	0.11	-7.87*	3.29	-0.97	1.46
Climatic demands (β_4)	0.00^{+}	0.00	-0.14	0.09	0.01	0.04
Human-made disaster (β_5)	-0.02	0.04	0.52	1.29	0.36	0.59
Pathogen x GDP (β_6)	-0.12^{+}	0.06	-3.22	1.98	-1.78*	0.89
Climate x GDP (β_7)	0.00	0.00	0.01	0.06	-0.04	0.03
Human-made disaster x GDP (β_8)	0.00	0.02	-0.09	0.72	0.11	0.33
Country variance (σ_e^2)	0.190		185.896		42.063	
Region variance (σ_u^2)	0.053		47.522		4.039	
ICC	0.217		0.204		0.088	
AIC	219.801		1251.727		1018.966	

Notes. $^{+}p < .10$; $*p < .05$; $**p < .01$; $***p < .001$

Table 9
Replications for All Multilevel Models Based on Maddison's Historical GDP data in 1950

Predictors	*Maddison's Historical GDP 1950*					
	Unified Democracy Score		Press Freedom		Economic Freedom	
	Estimate	*SE*	*Estimate*	*SE*	*Estimate*	*SE*
Intercept (β_0)	0.36*	0.18	54.90***	5.21	53.07***	2.43
ln GDP per capita (β_1)	0.40***	0.08	7.48**	2.37	3.61**	1.12
ln Population density (β_2)	0.03	0.04	0.92	1.20	2.22***	0.56
Historical pathogen prevalence (β_3)	-0.45***	0.13	-11.25**	3.78	-5.52**	1.82
Climatic demands (β_4)	0.00	0.00	0.00	0.11	0.00	0.05
Natural disaster casualty (β_5)	-0.02	0.03	-1.24	0.84	-0.60	0.39
Pathogen x GDP (β_6)	-0.01	0.13	-2.30	3.71	-5.43**	1.82
Climate x GDP (β_7)	0.00	0.00	-0.02	0.11	-0.13*	0.06
Disaster x GDP (β_8)	-0.03	0.04	-0.65	1.08	0.98	0.52
Country variance (σ_e^2)	0.230		202.207		51.732	
Region variance (σ_u^2)	0.035		29.168		2.298	
ICC	0.132		0.126		0.043	
AIC	189.755		961.957		799.721	
N	114		114		114	

Notes. *p < .05; **p < .01; ***p < .001

Table 10

Replications for All Multilevel Models Based on Maddison's Historical GDP data in 1929

Predictors	Maddison's Historical GDP 1929					
	Unified Democracy Score		Press Freedom		Economic Freedom	
	Estimate	*SE*	*Estimate*	*SE*	*Estimate*	*SE*
Intercept (β_0)	0.94**	0.26	54.43***	7.26	62.26***	3.94
ln GDP per capita (β_1)	0.08	0.16	0.69	5.07	-2.90	2.76
ln Population density (β_2)	-0.01	0.06	2.61	1.66	0.47	0.90
Historical pathogen prevalence (β_3)	-0.09	0.17	-3.82	5.34	-7.19*	2.90
Climatic demands (β_4)	0.01	0.00	0.07	0.14	-0.04	0.08
Natural disaster casualty (β_5)	-0.11**	0.04	-3.48**	1.15	-0.46	0.63
Pathogen x GDP (β_6)	-0.26	0.24	-11.99	7.72	-10.62*	4.19
Climate x GDP (β_7)	0.02**	0.01	0.41*	0.20	0.07	0.11
Disaster x GDP (β_8)	0.06	0.05	2.67	1.71	2.15*	0.93
Country variance (σ_e^2)	0.122		144.180		42.589	
Region variance (σ_u^2)	0.030		0.000		0.000	
ICC	0.199		0.000		0.000	
AIC	66.706		412.447		351.473	
N	50		50		50	

Notes. *$p < .05$; **$p < .01$; ***$p < .001$
